# Muscle activity relationships during isometric shoulder internal and external rotation using the ForceFrame dynamometer and athletic shoulder tests in baseball athletes

**DOI:** 10.3389/fphys.2025.1632248

**Published:** 2025-09-30

**Authors:** Ben Ashworth, Mikulas Hank, Omid Khaiyat, Ginny Coyles, Petr Miratsky, Ferdia Falon Verbruggen, Frantisek Zahalka, Tomas Maly

**Affiliations:** ^1^ School of Health and Sport Sciences, Liverpool Hope University, Liverpool, United Kingdom; ^2^ Sport Research Centre, Charles University, Prague, Czechia

**Keywords:** isometrics, muscular strength, performance, optimization, injury prevention

## Abstract

**Background:**

Optimal shoulder and trunk neuromuscular coordination significantly contributes to performance and injury prevention in overhead athletes. Although isolated shoulder rotation tests are often discussed in research, they may not fully evaluate the complex muscle synergies necessary for functional thrower positions or force deficiencies. Despite the increasing use of the athletic shoulder (ASH) test in elite sports, evidence regarding the relationship between muscle activation patterns in this multi-joint test and traditional isolated assessments is lacking. This study aimed to examine the muscle activity relationships during shoulder strength assessment of isometric rotation and the ASH test in overhead throwing athletes.

**Methods:**

Surface electromyography was used to analyze the muscle activation of nine shoulder and trunk muscles during maximal voluntary contractions in 17 male national team baseball athletes. Normalized values were used in muscle activation relationship analysis between internal/external rotation and ASH test’s three shoulder positions in the dominant arm (ASH I, ASH Y, and ASH T).

**Results:**

Analysis between internal rotation, external rotation, and the ASH I, ASH Y, and ASH T test positions showed significant differences in muscle activity (p < 0.05). The infraspinatus, anterior and posterior deltoid, and upper trapezius demonstrated higher activity in the shoulder rotation tests, while the external obliques and pectoralis major indicated higher activity during ASH tests. Moderate (ρ = 0.40–0.69) correlations were found between muscles during the tests, while strong (ρ = 0.70–0.89)-to-very strong (ρ = 0.90–1.00) correlations were found between individual muscles.

**Conclusion:**

The ASH and shoulder rotational tests’ results showed different shoulder and trunk neuromuscular activation levels, demonstrating a low relationship between the prime movers for ASH positions and muscles activated during shoulder rotation. This implies that muscle synergies vary significantly depending on joint positioning and test mechanics. It also emphasizes the importance of using multiple test batteries to capture force production deficits and abnormalities that are relevant to overhead athletes. The results support rehabilitation progression starting with infraspinatus strengthening in neutral shoulder abduction, followed by scapular stabilizer training at wider abduction angles to restore functional overhead capacity. Acute or chronic performance may be monitored weekly for further training optimization, return-to-sport decisions, and injury prevention in elite overhead throwing athletes.

## 1 Introduction

Field-based shoulder strength assessment seems optimal for coaches to monitor athletes’ performance in real-time, possibly showing acute or chronic (longitudinal) reactions to training volume/intensity and revealing fatigue or abnormal adaptation (Tremblay et al., 2024; Stone and Schilling, 2020; Erickson et al., 2017). This is essential for training optimization and injury prevention in elite overhead athletes (Camp et al., 2017). In baseball pitchers, maximal force transfer across the kinetic chain is crucial (Fleisig et al., 1996; Roach et al., 2013; Stodden et al., 2005). With ball speeds exceeding 140 km/h (90 mph) and shoulder angular velocities exceeding 6,000°/s, athletes rely on optimal coordination between the scapular and glenohumeral muscle complex, along with lower limb and trunk musculature (Seroyer et al., 2010; Digiovine et al., 1992; Escamilla and Andrews, 2009). Based on the evidence of throwing performance, weakness in shoulder external rotator muscles can lead to shoulder injuries (Asker et al., 2019). Additionally, the repetition of maximal throws may lead to muscle fatigue or imbalance in synergies, resulting in compensatory mechanisms that reduce performance and elevate injury risk (Sonne and Keir, 2016). The commonly used isometric strength assessments in overhead sports are predominantly focused on shoulder internal rotation (IR) and external rotation (ER) (Job et al., 2024; Couch et al., 2021). Rotation tests are performed in the supine position, evaluating short-lever IR and ER strength at 90° shoulder abduction and 90° elbow flexion (Couch et al., 2021; Cools et al., 2016). Although isolated shoulder strength tests are widely used in sports, they may not capture the complex muscle coordination or force production deficits relevant to overhead athletes. The athletic shoulder (ASH) test (Ashworth et al., 2018) was specifically developed to evaluate long-lever shoulder flexion by testing the isometric force in prone position in three shoulder positions, mimicking the demands of overhead actions and thus providing extra information than shoulder rotation testing in isolation (Ashworth et al., 2018). The positions are ASH I (180° shoulder abduction), ASH Y (135° shoulder abduction), and ASH T (90° shoulder abduction). Integration of the ASH test with tests of rotator cuff strength provides a valuable normative reference for practitioners, coaches, and athletes (Kadlec et al., 2020).

In baseball pitching, shoulder electromyographical (EMG) activity by muscle varies depending on the throwing phase, but due to motion complexity, the scapular and glenohumeral muscles behave synergistically and depend on agonist/antagonist requirements for kinetic transfer between the posterior chain (e.g., trunk extensors and scapular stabilizers) and the anterior chain (e.g., pectoralis major, internal shoulder rotators, or internal and external obliques) (Escamilla and Andrews, 2009; Fleisig et al., 2011). For example, the deltoids, infraspinatus, and teres minor show high activation during the stride and late-arm “cocking” phase, while the pectoralis major, latissimus dorsi, and the upper third of the subscapularis show high activation during early-arm acceleration. Additionally, elevated activity is present in the latissimus dorsi, lower trapezius, and posterior deltoid during shoulder horizontal flexion in late acceleration immediately prior to ball release and arm deceleration phases (Escamilla and Andrews, 2009). Further research showed that the external obliques show higher activation due to trunk rotational stability and force transmission in throwing activities involving the anterior oblique sling (Aoyama et al., 2022; Roach and Lieberman, 2014; Urquhart and Hodges, 2004; Owens et al., 2024). The important contribution of the lower body during the throw is also well-supported by EMG research findings (McMullen and Uhl, 2000; Oliver et al., 2015; Chu et al., 2016; Seroyer et al., 2010; Escamilla and Andrews, 2009). In contrast, this research focuses on the key roles of the shoulder and trunk, specifically including the ipsilateral and contralateral oblique muscles, in trunk stabilization and coordination of force transfer from the lower half to the shoulder during throwing (Conte et al., 2012; McMullen and Uhl, 2000). Previous studies have shown that shoulder muscle activation is highly position-dependent (Hecker et al., 2021; Alizadehkhaiyat et al., 2015). For example, infraspinatus activity typically decreases at higher abduction angles, while serratus anterior and trapezius activity increases, showing the different contributions of scapular stabilizers (Uga et al., 2016). Similarly, EMG analysis has demonstrated direction-specific differences between ER and IR rotation tasks, further suggesting that the test configuration affects the muscle strategy (Boettcher et al., 2010). The infraspinatus and subscapularis are important in IR and ER, while also stabilizing the glenohumeral joint during dynamic movements (Escamilla et al., 2012). Due to their higher physiological cross-sectional area, these muscles generate a high force (often exceeding that of the supraspinatus), especially during scapular plane abduction and rotational movements (Hik and Ackland, 2018). In addition to the ER, a heightened importance of the infraspinatus compared to supraspinatus in shoulder abduction based on their anatomical footprints has been proposed, supporting the concept that infraspinatus weakness may lead to supraspinatus injury (Mochizuki et al., 2008). Exercises involving the ER and IR at both 0° and 90° shoulder abduction effectively target these muscles, and their activation patterns can be optimized through proper scapular positioning to reduce injury risk and enhance performance (Escamilla et al., 2012). Additionally, conscious correction of scapular positioning during ER exercises enhances upper trapezius activation without altering the beneficial muscle ratios (De Mey et al., 2013). Overhead athletes with shoulder pain exhibit elevated activity in thoracohumeral and abdominal muscles during rotational movements, showing the role of trunk activation and compensatory strategies (Werin et al., 2020). These findings support the clinical relevance of assessing IR and ER strength in overhead athletes in functional positions.

The EMG research found high variability in shoulder muscle firing patterns between the subjects (e.g., inter-subject differences of the activation strategies); thus, assessing correlation patterns between muscles may offer an understanding of each individual’s neuromuscular coordination strategies between performance of various tasks (Schober et al., 2018). Despite the frequent use of IR and ER strength assessments and the recent rise in the use of ASH tests in overhead athlete profiling, no research has compared their muscle activation profiles and neuromuscular coordination patterns. While the peak force and rate of force development have been explored in the ASH test, current literature lacks evidence on how different test shoulder positions and movement directions affect the relationships between the shoulder and trunk muscles and how it differs between the IR and ER. This is important due to the differences in joint positioning, lever arm length, and muscular involvement (Ashworth et al., 2018; Trunt et al., 2022). This limitation creates a research gap, which may influence the clinical and performance understanding of using these tests and their interpretation in throwing sports associated with high injury risk. The current study addresses this gap by examining the EMG relationships between these commonly used tests to identify specific muscle synergies, offering practical insights for performance monitoring, injury prevention, and return-to-play decisions in baseball athletes. The question is, to what extent do various shoulder tests show different patterns of muscle activation and neuromuscular coordination? Identification of specific neuromuscular differences or synergies between the posterior and anterior shoulder and trunk may facilitate a deeper understanding of the coordination between various tests. Thus, we aimed to examine the EMG of shoulder and trunk muscle activity relationships during isometric strength assessment of shoulder rotation and ASH test in overhead athletes. The hypothesis was that the ASH test would indicate a higher activation of the anterior chain (pectoralis major, anterior deltoid, and external obliques) than the rotation tests and that different correlation patterns would indicate task-specific neuromuscular synergies between tests. Understanding these relationships will help coaches in distinguishing performance abnormalities, profiling and fatigue monitoring during the season, individual rehabilitation markers, and return-to-play decisions for overhead athletes (Gaudet et al., 2019; Cohen et al., 2022).

## 2 Materials and methods

### 2.1 Study design

This study used a cross-sectional design. Every participant was fully informed about the data collection and research procedures. All participants were informed of the experimental procedures and risks and had to provide a written informed consent prior to testing. The study was conducted in accordance with the guidelines of the Declaration of Helsinki. The ethics review board of the Institutional Ethical Committee of the Liverpool Hope University approved the study protocol.

### 2.2 Participants

The study analyzed a total of 17 baseball athletes (age, 22.7 ± 4.2 years; body height, 186.3 ± 7.3 m; body mass, 83.9 ± 10.1 kg). The participants were active members of the Czech Republic national team, including five position players and 12 pitchers. Only the dominant upper limb was selected as the preferred throwing arm by verbal questioning. The inclusion criteria included male baseball athletes who had at least 6 years of baseball training in the highest national competition and were at least 2-year members of the national team. The participants were excluded from the study if they reported any musculoskeletal injuries or signs of pain that would exclude them from active training or competition. Only data from participants who completed all five tests were used for analysis (Table 1). Participants were advised to avoid any high-intensity physical activity within 48 h prior to the testing session and were familiarized with the test protocol by asking them to perform a minimum of three complete tests on separate days prior to the study.

**TABLE 1:** Global summary of differences between %MVC and different testing conditions over the repeated measurements.

Muscle	Participants	Chi^2^	df	p-value
Upper trapezius	12	19.7657	4	0.0006
Posterior deltoid	15	43.4916	4	<0.0001
Anterior deltoid	15	28.6933	4	<0.0001
Infraspinatus	15	42.4698	4	<0.0001
Latissimus dorsi	15	18.1074	4	0.0012
Serratus anterior	14	41.8857	4	<0.0001
Pectoralis major	14	32.4	4	<0.0001
Right external oblique	16	23.8616	4	0.0001
Left external oblique	15	19.5987	4	0.0006

### 2.3 Procedures

#### 2.3.1 Surface EMG acquisition and analysis

**FIGURE 1:**
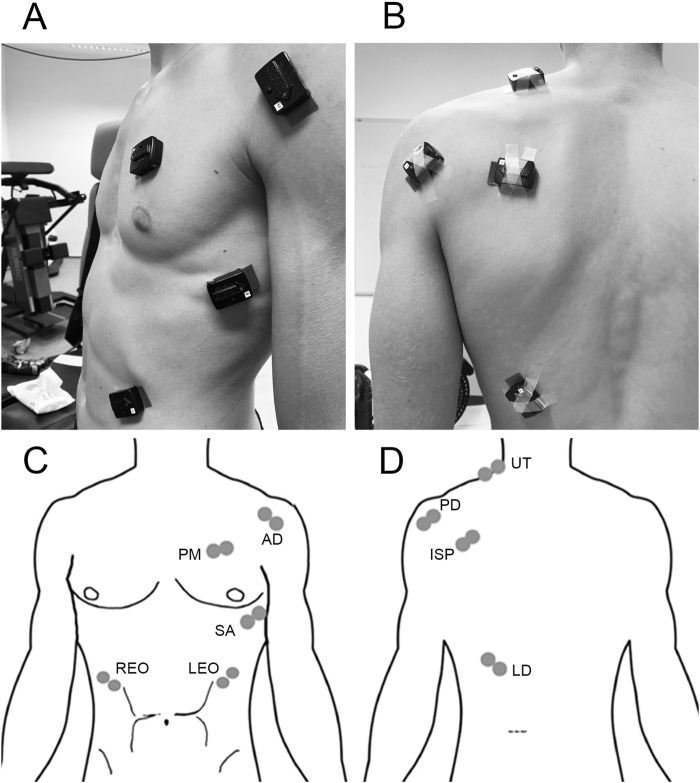
Electrode placement: **(A)** diagram of the front view of electrode placement; **(B)** diagram of the back view of electrode placement; **(C)** front view of electrode placement: anterior deltoid (AD), pectoralis major (PM), serratus anterior (SA), right external oblique (REO), and left external oblique (LEO); **(D)** back view of electrode placement: upper trapezius (UT), posterior deltoid (PD), infraspinatus (ISP), and latissimus dorsi (LD) (Alizadehkhaiyat et al., 2015).

Wireless surface electrodes (Trigno, Delsys Inc., Natick, United States) in accordance with EMGworks acquisition and analysis software (Trigno, Delsys Inc., Natick, United States; version 4.8.0) were used for signal acquisition, processing, and analysis from the nine shoulder and trunk muscles, and they were positioned to replicate established protocols (Owens et al., 2024): upper trapezius, anterior and posterior deltoid, infraspinatus, latissimus dorsi, serratus anterior, pectoralis major, and left and right external oblique muscles (Figure 1). The surface wireless sensor was placed on the selected area by an experienced physiotherapist (laboratory member). The area was first cleaned, shaved, and cleaned again with medical alcohol wipes (Medipal, alcohol wipes). The sensor was directly attached to the measurement position by double-sided adhesive manufacturer`s stickers and tape to prevent the loss of signal during the tests (Delsys Inc., Natick, United States). Surface EMG activity was recorded simultaneously in all tests with the sample rate at 2,048 Hz (Backus et al., 2010). A bandwidth of EMG signal was subjected to a high-pass filter (fourth-order Butterworth) at 45 Hz and low-pass filter (fourth-order Butterworth) at 450 Hz for further analysis (Navarro et al., 2023). The root mean square (RMS) non-overlapping window size of 20 ms was used for rectifying and smoothing the signal. RMS value of %MVC for each muscle was used to normalize signals. The standard frame interval was set at 0.0135 s.

#### 2.3.2 Normalization %MVC procedure

**FIGURE 2:**
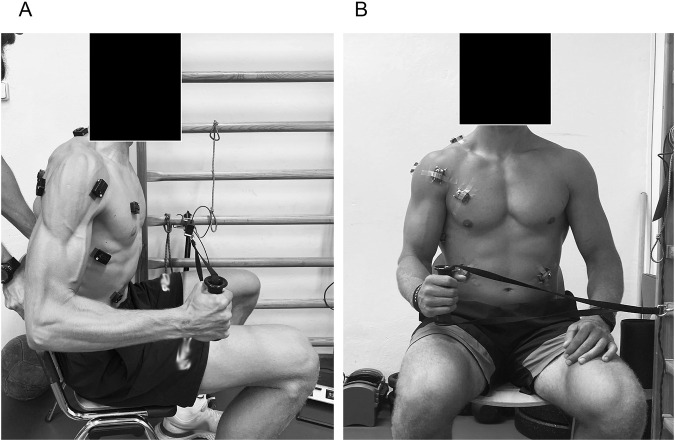
Percent maximal voluntary contraction (%MVC) strength assessment of shoulder internal and external rotation using a force gauge connected to a digital analyzer (MIE medical research ltd., Leeds, United Kingdom) in the seated body position with neutral 0° shoulder abduction and 90° elbow flexion. **(A)** Side view of the normalization %MVC procedure, **(B)** Front view of the normalization %MVC procedure.

To facilitate comparison between muscles, surface EMG data were normalized using %MVC using a force gauge connected to a digital analyzer (MIE medical research ltd., Leeds, United Kingdom) (Figure 2). The post-processing method used a maximum RMS value from each recording to normalize the subsequent EMG data series. The output was displayed as a percentage value (%MVC). For muscles producing ER moments at the shoulder (Hik and Ackland, 2018; Lieber and Ward, 2011), subjects performed the maximum normalized ER force while seated, with the dominant shoulder in neutral abduction and the elbow flexed to 90° (Cools et al., 2014). EMG data were collected for 3 s during each of three maximal trials, with the highest force repetition used as the reference value for normalization of EMG amplitudes during all tests for the posterior deltoid, infraspinatus, and serratus anterior. For muscles producing IR moments, normalization was performed using the maximum normalized IR force under the same conditions. The surface EMG recorded was used as a reference value for normalization of EMG amplitudes during all tests for the upper trapezius, anterior deltoid, pectoralis major, latissimus dorsi, and left and right external oblique muscles, which can be used to easily establish a reference for comparison between subjects (the mean IR force was 22.4 kg). This approach ensures consistent comparison of the relative activation levels across muscles and test types.

#### 2.3.3 Isometric shoulder rotation strength assessment

**FIGURE 3:**
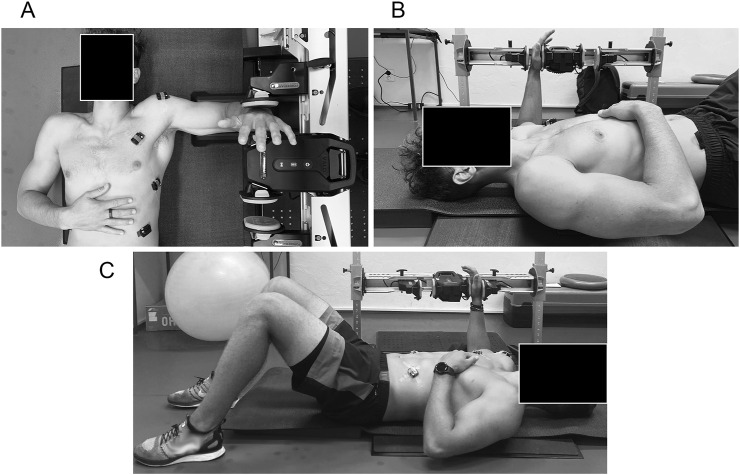
Percent maximal voluntary contraction (%MVC) strength assessment of shoulder internal and external rotation using an isometric dynamometer (ForceFrame, Vald, Australia) in the supine body position with 90° shoulder abduction and 90 elbow flexion. **(A)** Top view of the strength assessment of shoulder internal and external rotation. **(B)** Right view of the strength assessment of shoulder internal and external rotation. **(C)** Left view of the strength assessment of shoulder internal and external rotation.

IR and ER shoulder strength assessment was performed using an isometric dynamometer (ForceFrame, Vald, Australia). In addition to individual shoulder mobilization warm-up, participants performed a standardized exercise of 3 × 10 repetitions (each side) of shoulder IR and ER with a medium-resistance elastic band in the standing position. For testing, the body was positioned supine with 90° abduction and 90° elbow flexion, with the knees flexed and feet flat on the floor, and contralateral arm on rib cage and arm abduction set at 90° (Figure 3). The elbow was supported in a custom-designed foam sleeve (Vald, Australia) to standardize the test position and minimize compensations such as unwanted shoulder adduction or abduction. The participant’s hand was placed such that the heel of the hand was positioned at a comfortable height to push into the load cell. For familiarization, participants performed three submaximal trials of 3 s separated by a 30-s rest period. The %MVC protocol consisted of three maximal 3-s trials (30 s between reps) of IR first, followed by ER in the dominant upper limb separated by a 60-s rest period. EMG signals were recorded within every %MVC repetition.

#### 2.3.4 Isometric shoulder flexion strength assessment

**FIGURE 4:**
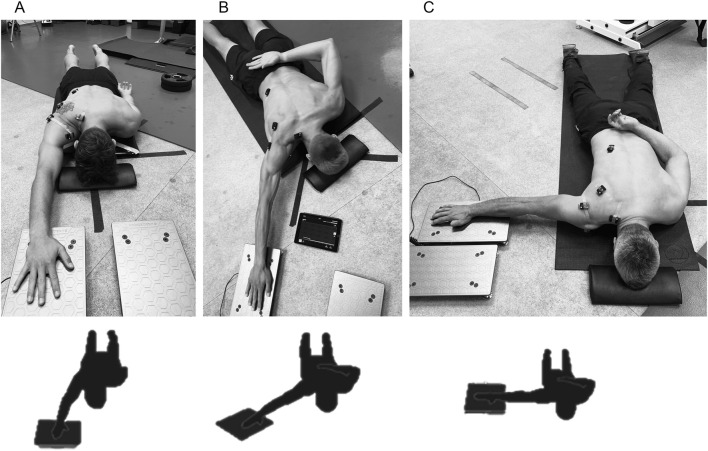
Percent maximal voluntary contraction (%MVC) strength assessment of shoulder flexion by athletic shoulder (ASH) test using a force plate system (ForceDecks, Vald, Australia) in the prone body position with a fully extended elbow in three shoulder abduction positions: **(A)** ASH I (180° shoulder abduction); **(B)** ASH Y (135° shoulder abduction); **(C)** ASH T (90° shoulder abduction).

The ASH test was conducted following the original protocol (Ashworth et al., 2018). The participants performed maximal isometric contractions in three shoulder positions: ASH I (180° shoulder abduction), ASH Y (135° shoulder abduction), and ASH T (90° shoulder abduction), and the forearm was pronated, with the heel of the hand as the primary contact point on the force platform (Figure 4). In all three positions, the elbow was as fully extended as possible/comfortable, and the scapula was maintained in a natural position relative to the elevated arm. All tests were carried out with the participant lying prone on the floor, with their forehead resting on a 4-cm foam block to standardize the neck position. The contralateral arm was placed behind the back during ASH Y and ASH T tests to enhance trunk stability. For the I-test, the contralateral arm remained by the participant’s side due to lower rotational forces encountered. No part of the subjects’ forearm was allowed to rest on the force platform, and consistent force application through the heel of the hand was required for testing. A vertical-axis force plate system (ForceDecks, Vald, Australia) connected to proprietary data acquisition and analysis software was used to measure the force output. The platform was positioned on the floor adjacent to the participant’s shoulder. After a standardized warm-up consisting of the individual players’ throwing warm-up, followed by two submaximal (80%–90% physical effort) contractions in each test position, the participants performed three maximal 3-s trials in each position on the dominant limb separated by a 30-s rest period. The order of testing was consistently ASH I, ASH Y, and ASH T, as recommended by the original protocol (Ashworth et al., 2018). The participants received standardized instructions (Maffiuletti et al., 2016) and consistent verbal encouragement during each trial, including “push as fast and hard as possible.” A verbal countdown was provided prior to each effort. Trials were excluded and repeated if there was an instance of a failure to perform the test according to the instructions.

### 2.4 Statistical analysis

Due to violations of normality assumptions confirmed by the Shapiro–Wilk test (p < 0.05 for all muscles), descriptive statistics for each muscle and test are presented as medians with interquartile range of the 25th and 75th percentile. A nonparametric repeated-measure ANOVA was performed using Friedman test to evaluate within-subject differences in muscle activity (%MVC; dependent variable) across five isometric strength tests (independent variable). Wilcoxon signed-rank *post hoc* test with Bonferroni correction was used to evaluate pairwise comparisons. For maximal statistical clarity, only data from participants who completed all five tests were used for statistical comparison. Muscle activation levels were classified according to prior studies (Digiovine et al., 1992) as low (0%–20% MVC), moderate (21%–40% MVC), high (41%–60% MVC), and very high (above 60% MVC). To examine relationships between muscle activation patterns across tests, pairwise Spearman’s correlation coefficients (ρ) were calculated and scaled as negligible (0.00–0.10), weak (0.10–0.39), moderate (0.40–0.69), strong (0.70–0.89), and very strong (0.90–1.00) (Schober et al., 2018). A significance level of p < 0.05 was used for all statistical tests. All statistical analyses were performed using IBM® SPSS® v21 (Statistical Package for Social Science, Inc., Chicago, IL, 2012) and Python (v 3.12.3; Python Software Foundation) for data processing, visualization, and inferential testing.

## 3 Results

**FIGURE 5:**
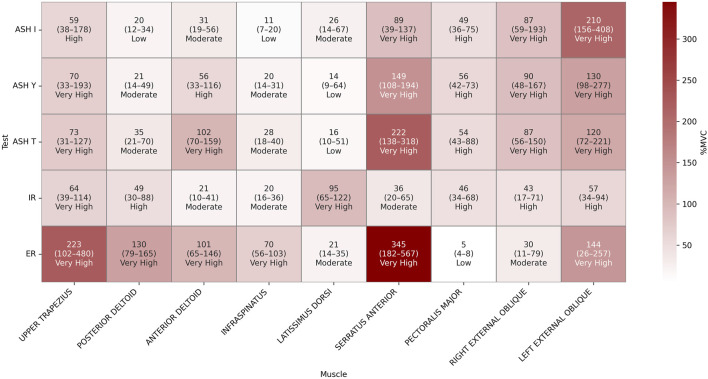
Heatmap of percent maximal voluntary contraction (%MVC) descriptive results presented as the median (25th–75th percentile) values for each muscle across five isometric strength tests: athletic shoulder test (ASH) in three shoulder positions (ASH I, ASH Y, and ASH T) and shoulder internal rotation (IR) and shoulder external rotation (ER).

Muscles reached relatively high maximum activations from 150 up to 500 %MVC in individual cases, such as the serratus anterior or upper trapezius in ER (Figure 5). High median values ranged from 200 to 350 %MVCs, particularly the median serratus anterior activity in ER (over the 300 %MVC) and ASH T (over 200 %MVC). The upper trapezius showed values above 200 %MVC in ER. The last muscle that reached the median over 200 %MVC was the left external obliques in ASH I. The rest of the muscles showed values between 150 and 100 %MVC or less.

**FIGURE 6:**
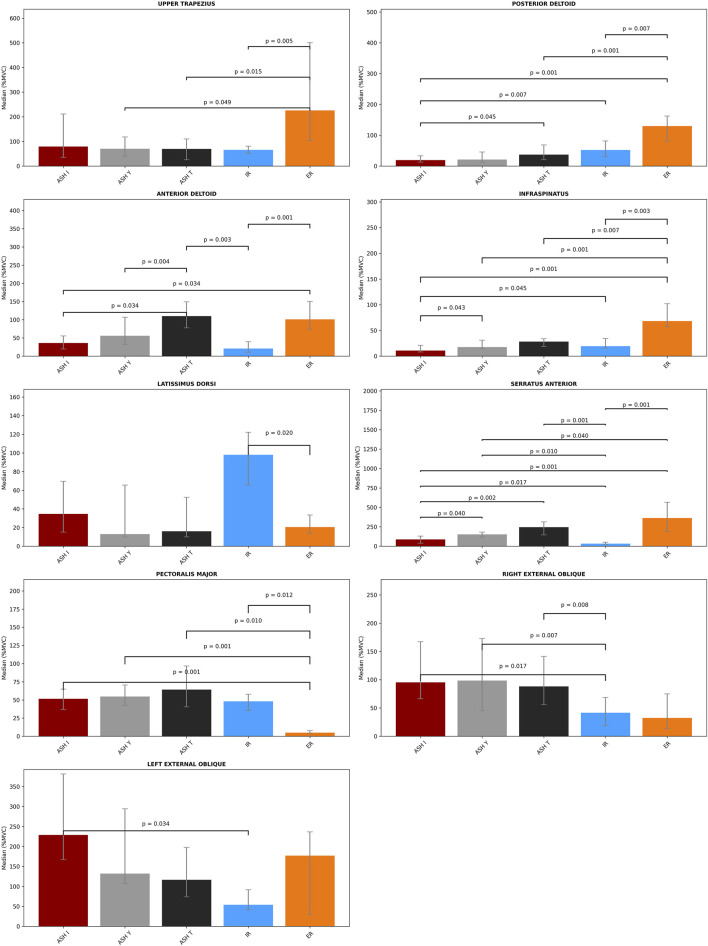
Bar plots with the median values and error bars (interquartile ranges between 25th and 75th percentile) of percent maximal voluntary contraction (%MVC) for each muscle across five isometric strength tests: athletic shoulder test (ASH) in three shoulder positions (ASH I, ASH Y, and ASH T) and shoulder internal rotation (IR) and shoulder external rotation (ER). Pairwise statistical significance (Wilcoxon tests with Bonferroni correction) is shown above the interconnection lines by the p-value.

The findings of a repeated-measure ANOVA indicated a significant main effect of the test condition across all the analyzed muscles (Table 1). Significant results of *post hoc* pairwise comparisons and exact p-values are shown in Figure 6. The highest number of significant differences (p < 0.05) to other tests within their muscle activations was found in ER, particularly for the infraspinatus and pectoralis major. The posterior and anterior deltoids also recorded a higher number of different (p < 0.05) EMG activities between tests. By contrast, muscles with the most differences between tests were the infraspinatus and serratus anterior. For the infraspinatus, no difference (p > 0.05) was found only between ASH T vs. ASH I/ASH Y and IR vs. ASH I/ASH Y. For the serratus anterior, no difference (p > 0.05) was found only between ASH T vs. ASH Y/ER.

From the perspective of reaching 60% significantly higher activations, the external shoulder rotation test ER activated the serratus anterior approximately 330 %MVC higher (p < 0.05) than in IR and was additionally approximately 276% higher (p < 0.05) than in ASH I and 211% higher (p < 0.05) than in ASH Y. Another highly elevated activation by ER was in observed in the upper trapezius, which differed approximately 160% (p < 0.05) when compared to IR and almost 160% (p < 0.05) in ASH T or ASH Y. ER also raised the activity of the posterior deltoid by approximately 100% higher (p < 0.05) than in ASH I and ASH T and of the anterior deltoid by 80% when compared to IR, 74% more than in ASH I (p < 0.05) and 65% more than in ASH I. Otherwise, the latissimus dorsi reached almost 80% higher results in IR than in ER. The infraspinatus was found to demonstrate higher ER activation (p < 0.05) when compared to ASH tests by 40%–60%.

One of the highest differences within ASH tests was the EMG activity of the serratus anterior, which was particularly 159% higher (p < 0.05) in ASH T than in ASH I and 66% higher in ASH Y vs. ASH I. Additionally, a difference was found in the anterior deltoid when ASH T reached 74% more (p < 0.05) than ASH I. Interestingly, the left external oblique reached an approximately 100% higher median value in ASH I than in ASH Y and T, but with no statistical difference (p > 0.05, respectively).

When comparing ASH tests to rotational shoulder performance, the highest differences were found in the serratus anterior activity; ASH T reached 213% higher values and ASH Y reached 120% higher values than in IR (p < 0.05). ASH I excited the left external oblique 175% more and the ASH T anterior deltoid 90% more than IR (p < 0.05), respectively. Lower, but significantly higher (54%, p < 0.05) activation of the serratus was found in ASH I compared to IR.

Other differences of 40%–60% higher (p < 0.05) activations in ASH tests were evaluated mainly between the right external oblique and pectoralis major muscles compared to IR and ER. The pectoralis major reached 60% higher activation in ASH T compared to ER (p < 0.05). The right external oblique differed mostly in ASH Y (57% higher) and ASH I (54% higher) than in IR (p < 0.05).

**FIGURE 7:**
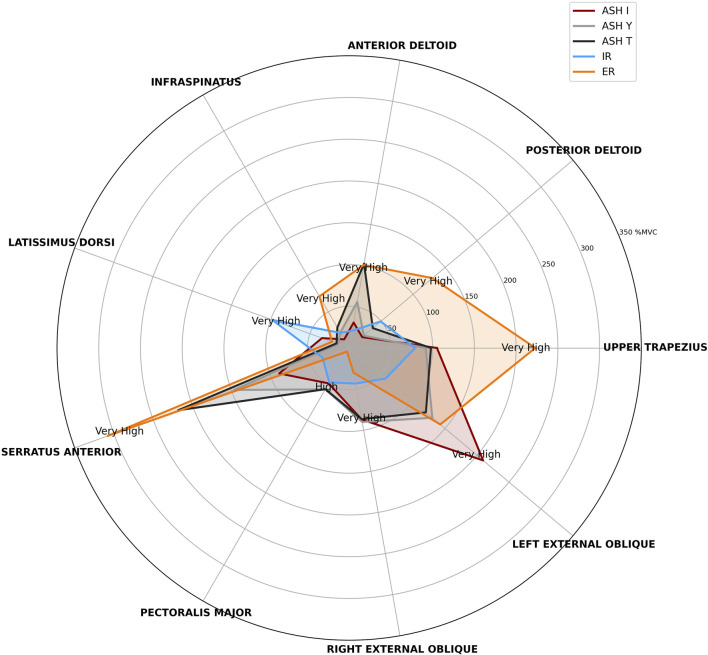
Radar plot of median percent maximal voluntary contraction (%MVC) for each muscle across five isometric strength tests: athletic shoulder test (ASH) in three shoulder positions (ASH I, ASH Y, and ASH T) and shoulder internal rotation (IR) and shoulder external rotation (ER). Activation magnitudes are classified into four levels (low <20%, moderate 20%–39%, high 40%–59%, and very high ≥60%), but only high and very high are shown in the graph.

**FIGURE 8:**
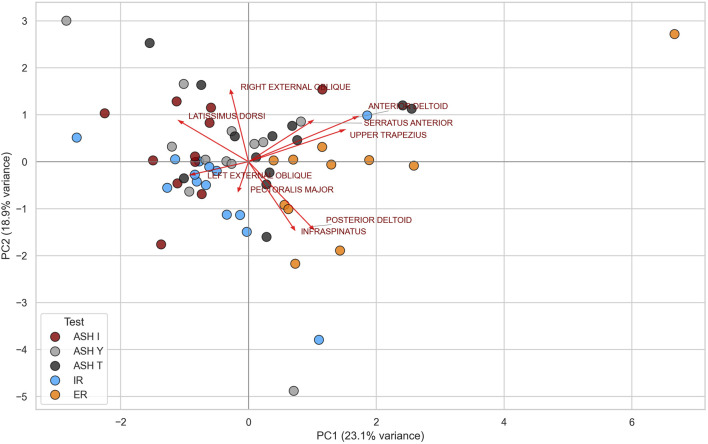
Principal component analysis (PCA) of global EMG activation patterns across five test conditions: athletic shoulder test (ASH) in three shoulder positions (ASH I, ASH Y, and ASH T) and shoulder internal rotation (IR) and shoulder external rotation (ER).

Owing to the specific clustering patterns emerging between muscle parts and different test types, descriptive data comprising activation magnitudes within all five tests with two-level classification (high 40%–59%; very high ≥60%) are shown in Figure 7. Additionally, evaluation of the principal component analysis (PCA) helps visualize the global patterns of potential neuromuscular coordination across test conditions (Figure 8). In general, PCA reduces the complexity of multivariate data through the identification of new composite variables (principal components that capture the most variance in data). The first principal component (PC1) represented the direction of the greatest variability among all EMG signals (the strongest overall pattern), while the second (PC2) captured the second largest variance, orthogonal to PC1. PC1 explained 23.1% of all the variations in muscle activation patterns, and PC2 explained 18.9%, which meant that together they explained 42% of the overall variability. Together, PC1 and PC2 explained 42% of the overall variability in the data, meaning that almost half of the variability across all muscle activation patterns and test conditions can be described by just two main patterns. This suggests that despite complex neuromuscular coordination, there are consistent activation strategies shared across participants and tests. The remaining variance probably reflects individual- and test-specific differences and natural variability that is typical during collecting EMG data.

**FIGURE 9:**
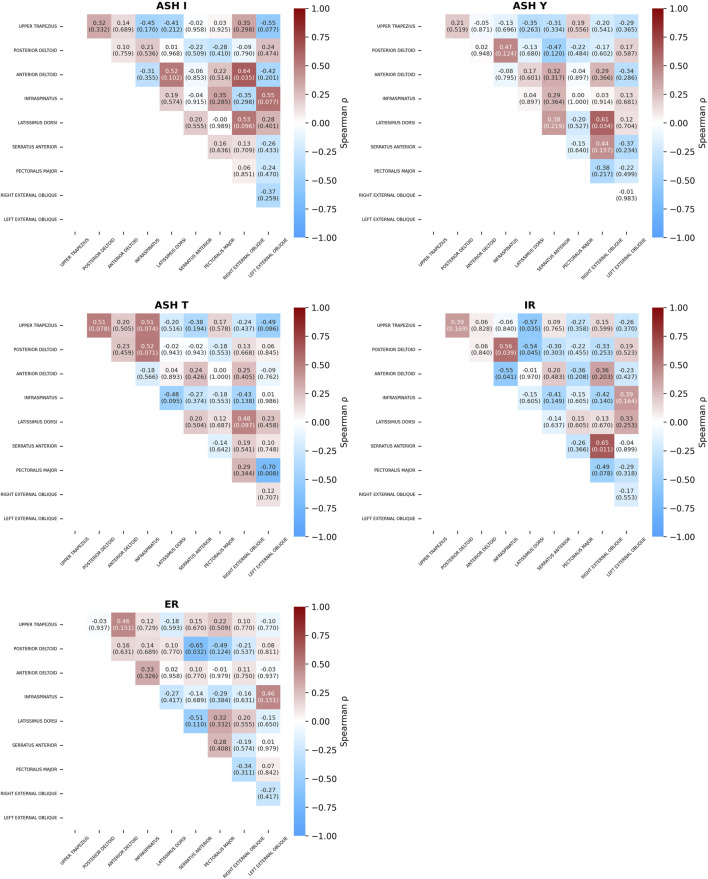
Spearman’s correlation coefficients (p-values) between muscle activity across five isometric strength tests: athletic shoulder test (ASH) in three shoulder positions (ASH I, ASH Y, and ASH T) and shoulder internal rotation (IR) and shoulder external rotation (ER).

Moderate positive significant correlations between individual muscles’ %MVCs were observed in several tests (Figure 9). Synergy between the anterior deltoid and right external oblique was found in the ASH I test (ρ = 0.64, p = 0.04). ASH Y showed a positive significant correlation between the latissimus dorsi and right external oblique (ρ = 0.61, p = 0.03). Moderate correlation in IR was found between the posterior deltoid and infraspinatus (ρ = 0.56, p = 0.04). The highest significant positive correlation was found in IR between the serratus anterior and right external oblique (ρ = 0.65, p = 0.01).

Moderately negative significant correlations were found in the ASH T test between the pectoralis major vs. left external oblique (ρ = −0.7, p = 0.01), while in ASH I test, moderate but non-significant (ρ = −0.55, p = 0.07) correlation was found between the upper trapezius vs. left external oblique. Another significant negative correlation was found in the IR test between the upper trapezius vs. latissimus dorsi (ρ = −0.57, p = 0.03), anterior deltoid vs. infraspinatus (ρ = −0.55, p = 0.04), and posterior deltoid vs. latissimus dorsi (ρ = −0.54, p = 0.04). ER resulted in negative synergy between the posterior deltoid vs. serratus anterior (ρ = −0.65, p = 0.03).

Moderate positive and negative correlations between ρ = 0.41 to 0.55 were found in ASH and rotation tests, but they were nonsignificant. Specifically, three moderate positive correlations were found in ASH I: infraspinatus vs. left external oblique (ρ = 0.55), latissimus dorsi vs. right external oblique (ρ = 0.53), and latissimus dorsi vs. anterior deltoid (ρ = 0.52). The ASH T test showed four moderate positive correlations: trapezius vs. posterior deltoid (ρ = 0.51), trapezius vs. infraspinatus (ρ = 0.51), posterior deltoid vs. infraspinatus (ρ = 0.52), and latissimus dorsi vs. right external oblique (ρ = 0.48).

Negative correlations were found between latissimus dorsi vs. serratus anterior (ρ = −0.51) and posterior deltoid vs. pectoralis major (ρ = −0.49) in ER and between pectoralis major vs. right external oblique (ρ = −0.49) in IR. The ASH T test showed three negative moderate correlations: upper trapezius vs. left external oblique (ρ = −0.49), infraspinatus vs. latissimus dorsi (ρ = −0.48), and infraspinatus vs. right external oblique (ρ = −0.43). Test AHI I resulted in negative correlations between the upper trapezius vs. infraspinatus (ρ = −0.45) and between the anterior deltoid vs. left external oblique (ρ = −0.42).

**FIGURE 10:**
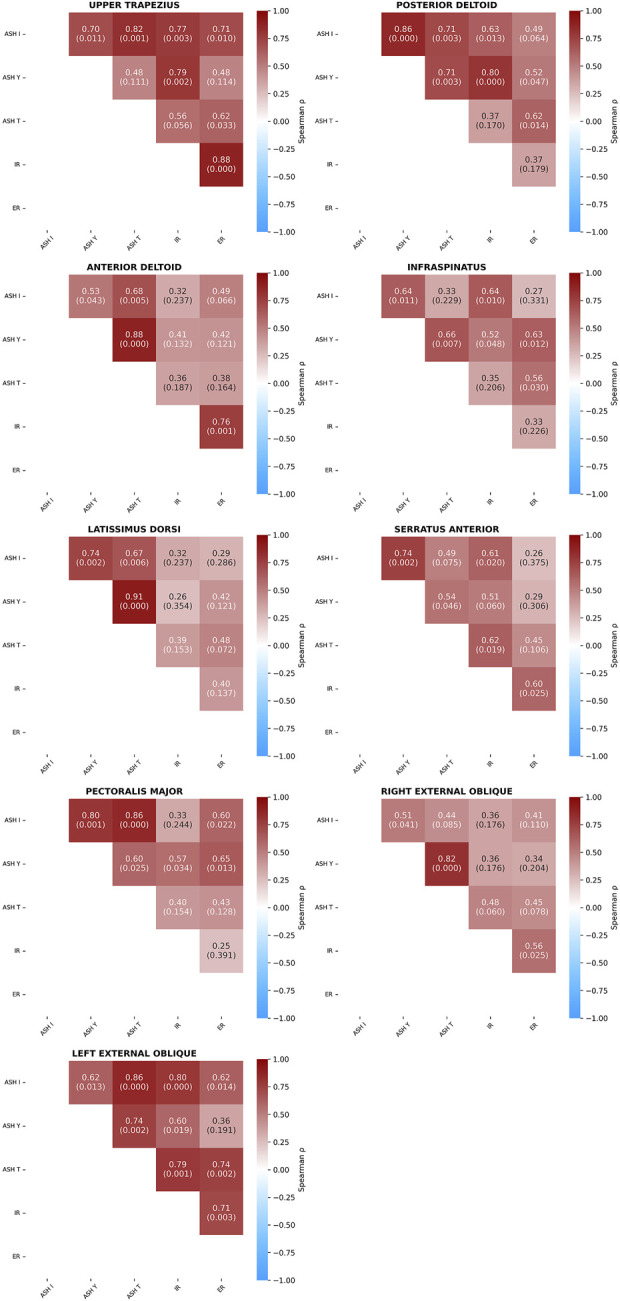
Spearman’s correlation coefficients (p-values) between five test conditions within individual muscles. Athletic shoulder test (ASH) in three shoulder positions (ASH I, ASH Y, and ASH T) and shoulder internal rotation (IR) and shoulder external rotation (ER).

Analysis of relationships between different testing protocols within individual muscles showed several pairs with significant positive correlations (Figure 10). A very strong relationship was found between ASH Y and ASH I for the latissimus dorsi (ρ = 0.91, p < 0.001), and the relationship was strong for the anterior deltoid (ρ = 0.88, p < 0.001). Four significant strong correlations were found between ASH T and ASH I tests for the left external oblique (ρ = 0.86, p < 0.001), pectoralis major (ρ = 0.86, p < 0.001), right external oblique (ρ = 0.82, p < 0.001), and upper trapezius (ρ = 0.82, p < 0.01). The left external oblique showed a relationship between ASH I vs. IR (ρ = 0.80, p < 0.001). Four other strong relationships (ρ = 0.77 to 0.80, p < 0.001) were found between the IR and ASH tests for the posterior deltoid (ASH Y), left external oblique (ASH T), and upper trapezius (ASH Y, ASH I).

## 4 Discussion

The aim of this study was to examine the relationships of shoulder and trunk muscle activations across five different isometric strength tests. Findings supported the hypothesis that different shoulder test positions (ASH I, ASH Y, ASH T, and IR/ER) activated specific neuromuscular synergies (muscle recruitment patterns and levels of activation) of the shoulder and trunk muscles differently. The most important finding was that the infraspinatus, posterior deltoid, and upper trapezius significantly contributed to the shoulder external rotation cluster (PC1). This suggests their predominant involvement in driving ER and differentiates their role in other tests. Conversely, the external obliques, latissimus, and pectoralis were oriented in the opposite direction, corresponding to their higher relative activity during more anteriorly and horizontally directed ASH test tasks. The relatively lower contribution of the posterior shoulder muscles during horizontal flexion and IR test directions supports previous EMG findings by Wattanaprakornkul et al. (2011), which reported that muscles such as the infraspinatus or posterior deltoid primarily function as dynamic stabilizers rather than prime movers during such movements. The results of this study similarly suggest that their role seems to be more supportive and postural during tests rather than reaching high levels of activation. Additionally, the levels of muscle EMG activation seem to have linear relationships with the force in isometric muscle actions, but not with isotonic, high-speed movements like throwing a baseball (Staudenmann et al., 2010; Escamilla and Andrews, 2009).

The differing patterns of muscle activation reflect unique neuromuscular demands, especially on the scapulo-humeral and thoraco-humeral stabilizers. In contrast, weak or test-specific correlations (e.g., between IR and ASH I) highlight how different joint positions may selectively influence the neuromuscular demand of trunk stabilizers, reinforcing the value of using an individual test battery in shoulder performance diagnostics. EMG has become a valuable tool to study muscle activation and coordination patterns, offering insights into recruitment strategies, intermuscular synergies during functional tasks, and neuromuscular fatigue (Cifrek et al., 2009; Navarro et al., 2023). In addition to traditional force measures in overhead athletes, EMG may measure the magnitude and timing of muscle activation across different test positions and evaluate functional performance (Owens et al., 2024; Alizadehkhaiyat et al., 2015). However, the interpretation of EMG is complex, and it is influenced by factors such as normalization methods, electrode placement, and muscle anatomy (Halaki and Ginn, 2012; De Luca et al., 2015). Valid normalization protocols, such as %MVC, are essential to compare data across muscles and conditions, especially when high-intensity contractions during sports actions may exceed standard %MVC levels (Burden and Bartlett, 1999). Previous research has described the direction-specific behavior of shoulder muscles (Boettcher et al., 2010; Uga et al., 2016), and findings of this research align with these observations. The infraspinatus demonstrated higher activation at lower shoulder elevations, while the serratus anterior and upper trapezius showed increased activity at higher abduction angles, especially during ASH Y and T. This “angle-specific” recruitment has implications for both testing and exercise prescription. The high variability in muscle activation values, especially in the anterior deltoid and serratus anterior across the test types, may reflect individual neuromuscular strategies or differences in muscle morphology and sensor placement. Despite normalization to %MVC, caution is warranted when interpreting %MVC across different muscles as 100 %MVC in one muscle does not necessarily equate to the same absolute functional output as another (Halaki and Ginn, 2012). In this study, a normalization approach using commonly standardized MVC test for IR and ER in 0° shoulder abduction and 90° elbow flexion, which showed acceptable reliability for clinical use, was used (Cools et al., 2014). However, research found that the participants’ body position, shoulder position, and equipment can influence the results (Job et al., 2024; Cools et al., 2014; Riemann et al., 2010). Single-IR and -ER tests for normalization in this research were chosen to reduce participant and laboratory schedule possibilities, maintain consistency across trials, and minimize fatigue. The commonly used seated position with 0° shoulder abduction was chosen to distinguish from the IR and ER shoulder positions of supine 90° shoulder abduction and 90° elbow flexion, which was used further in IR and ER assessments. Both positions were found to be confidently used to assess the shoulder rotation maximal forces; however, the supine position (90°) seems to be most consistent, and it is recommended for use by clinicians (Job et al., 2024; Cools et al., 2014). Additionally, higher levels of activation %MVC values found in the supine position (90° abduction) than when seated (0° abduction) supported the knowledge that the position may influence individual muscle recruitment to perform shoulder IR and ER. This emphasized how individual muscle activation may vary, especially in the serratus anterior, upper trapezius, posterior deltoids, and trunk rotators. However, it is acknowledged that this single global method may not capture the specific maximal activation capacity for all individual muscles tested, particularly for the mentioned trunk or scapular stabilizers such as the serratus anterior or external obliques. More specific muscle normalization procedures (such as resisted protraction for the serratus anterior or trunk rotation for the internal and external obliques) could improve the clinical value and should be considered in future research aiming for precise quantification of muscle activation levels.

In conclusion, the test position plays a critical role in shoulder muscle function, with 90° abduction being particularly relevant for assessing the force output and replicating joint angles observed during key throwing phases (Job et al., 2024; Escamilla et al., 2023; Cools et al., 2014). Hecker et al. (2021) demonstrated that shoulder muscle contributions are also highly dependent on the position. In their research, deltoid paralysis had no effect on IR strength but led to a 50% reduction in ER strength at 90° abduction, partly due to the involvement of the teres minor, given that both muscles are innervated by the same nerve. These findings highlight that both shoulder abduction and the rotation direction significantly influence force production. The deltoid’s role was shown to increase with greater abduction angles, further supporting its position-dependent contribution to shoulder strength (Hecker et al., 2021). Similar to the ASH test correlation with the volleyball serve (Del Águila et al., 20211), another study reported that IR torque of the shoulder in the abducted external rotated position (90° abduction and 90° external rotation, in a prone position using a hand-held dynamometer) was significantly correlated with racket velocity (r = 0.65; p < 0.05), concluding that shoulder IR strength in this position was a valid measurement for evaluating badminton players (Awatani et al., 2018).

The inclusion of trunk muscles, particularly the external obliques, proved significant activation differences between the tests. The contralateral external oblique is known to contribute to rotational stability and trunk force transmission during throwing (Urquhart and Hodges, 2004; Owens et al., 2024; Hank et al., 2024). Research data showed notably higher external oblique activation during ASH tests, emphasizing the test’s demand on trunk stabilization. Owens et al. (2024) showed an increase in contralateral external oblique muscle activity prior to throwing ball release, which was supported by Urquhart and Hodges (2004) who showed that contralateral external oblique causes and controls rotation during throwing, suggesting that the oblique muscles are an important part in overhead athletes’ performance evaluation and research.

Practical application and perspective from the results of this research has several positive implications. Regular athlete monitoring at relatively low costs using rotational and ASH tests is used by elite coaches and clubs to collect data on a weekly basis, contributing to optimized conditioning and subsequent performance. Early detection of abnormalities in the test performance after competition or prior to high-volume/-intensity practice may significantly reduce injury-related issues in combination with workload management. Another factor is evaluating the efficiency of adaptation/maladaptation of strength interventions or after high-repetition throwing in mainly the dominant arm. Testing both the dominant and non-dominant sides may uncover abnormal bilateral asymmetries or a global decrease in scores that are more indicative of more global causes of fatigue (Birfer et al., 2019; Erickson et al., 2016). Intersubject results may allow comparison between athletes, but intrasubject monitoring can establish “basic” thresholds for an individual athlete used to inform data-driven return-to-performance processes. The study confirms that infraspinatus activation is maximized in neutral shoulder abduction, making it a preferred position for early-phase rehabilitation following tendon injury or surgery (Uga et al., 2016). In contrast, training at 90° abduction (as in ASH T) elicits higher activation of scapular stabilizers such as the serratus anterior and synergistic muscles such as the anterior deltoid and pectoralis major, which are crucial for dynamic shoulder stability during throwing. Therefore, clinicians may use a progression from neutral to abducted positions to sequentially target isolated rotator cuff activation and then functional muscle synergies that are relevant to overhead athletes.

The limitation of this research was the relatively high variability in muscle activity across participants, which may be attributed to various factors. Coefficients of variation were notably high in several conditions, exceeding 100% in muscles such as the anterior deltoid during ER (CV = 109.3%) and ASH I (CV = 107.8%), reflecting substantial intersubject variability in neuromuscular strategies. Although we used an absolute selection of the available elite baseball players in the Czech Republic, we recommend performing sample-size estimation, which was the limit in our case. These findings are consistent with those of previous research in overhead athletes, where the variability reflects the diversity in neuromuscular recruitment strategies, functional adaptations, normalization of EMG procedures, individual neuromuscular characteristics, electrode placement, and typical EMG electrode conductivity issues (Halaki and Ginn, 2015). Another limitation was using only one global exercise for all muscles %MVC normalization, which may lead to biased and non-standard elevated values of specific muscle activities. This was mainly due to the laboratory and participants’ schedules and the relatively large numbers of tests performed and participants during the days. Future research should consider using specific %MVC test for individual muscles for normalization. The research revealed direction-specific and angle-specific relationships in muscle activity, with certain positioning increasing muscle activation in particular tests. However, the relatively small subject group, despite multiple repetitions, may have limited the statistical significance of the results. Increasing the number of subjects and repetitions could potentially enhance the study’s statistical power and generalizability. Additionally, performing randomized measurement positions would be recommended in future as not doing so may create a bias due to learning and fatigue effects, mainly in less experienced athletes or subjects.

Future studies should test how different familiarization periods or randomizing ASH test positions affect the results, include more high-level athletes, and repeat testing at multiple times during the season to track changes in athlete readiness. The limited number of EMG sensors prevented the evaluation of several muscles’ activities. Examination of additional significant shoulder and trunk muscles is recommended, such as the supraspinatus, subscapularis, rectus abdominis, or internal obliques. Consistent test protocols should be maintained. Additionally, linking these EMG findings to performance outcomes and injury data could enhance the practical application of ASH tests during training and rehabilitation.

## 5 Conclusion

The findings confirmed that different tests evoke specific higher neuromuscular activation and a low relationship between the primary movers of ASH tests and rotators, supporting that task-specific muscle synergies vary significantly depending on joint positioning and test mechanics. This emphasizes the importance of multiple test batteries in evaluating overhead athletes’ physical state and the current readiness to perform. Particularly, combining tests such as ASH and shoulder rotation to globally characterize shoulder function adds additional information about overhead athletes. The long-lever test positions mimicking aspects of baseball pitching appropriately challenge different muscle actions than more traditional rotational test protocols. The results support a rehabilitation progression starting with infraspinatus strengthening in neutral shoulder abduction, which is followed by scapular stabilizer training at wider abduction angles to restore functional overhead capacity. Acute or chronic performance may be monitored by coaches and clinicians on a weekly basis for training optimization, detection of abnormalities, rehabilitation planning, return-to-play decisions, and injury prevention in elite throwing populations.

## Data Availability

The raw data supporting the conclusions of this article will be made available by the authors, without undue reservation.
